# Intravenous contrast medium impairs CT-based muscle quality but not quantity assessment: a translational study

**DOI:** 10.3389/fradi.2026.1746296

**Published:** 2026-05-08

**Authors:** Luca Salhöfer, Gregor Jost, Mathias Holtkamp, Jannis Straus, Marcel Opitz, Sebastian Zensen, Rene Hosch, Johannes Harmes, Lale Umutlu, Michael Forsting, Felix Nensa, Hubertus Pietsch, Johannes Haubold

**Affiliations:** 1 Institute of Diagnostic and Interventional Radiology and Neuroradiology, University Hospital Essen, Essen, Germany; 2 Institute for Artificial Intelligence in Medicine, University Hospital Essen, Essen, Germany; 3 MR & CT Contrast Media Research, Bayer AG, Berlin, Germany

**Keywords:** body composition analysis, contrast medium, CT imaging, myosteatosis, opportunistic screening, sarcopenia, segmentation

## Abstract

**Objectives:**

CT-based body composition analysis (BCA) provides imaging biomarkers, including muscle volume and surrogates of muscle quality. Concerns over the comparability of Non-contrast and contrast-enhanced CT scans have limited their clinical application. This study aims to assess the influence of various contrast phases on a volumetric CT-based BCA.

**Materials and methods:**

20 Göttingen minipigs were subjected to a Non-contrast (NC) and five contrast-enhanced [Early Arterial, Late Arterial, Vascular Portal Venous, Parenchymal Portal Venous (PPV), Late] CT scans. 114 tri-phasic (Non-Contrast, Arterial, Venous) CT scans were analyzed for human validation. A volumetric BCA network [Body and Organ Analysis (BOA)] extracted muscle radiodensity and the following features as volumes: Muscle, Subcutaneous Adipose Tissue (SAT), Inter- and Intramuscular Adipose Tissue (IMAT), Visceral Adipose Tissue (VAT), and Total Adipose Tissue (TAT). Significance was assessed by a one-way ANOVA with Tukey's multiple comparisons test.

**Results:**

In the animal model, there was a tendency toward reduced IMAT volumes after CM injection [NC = 245 ml (±105 ml), e.g., PPV = 241 ml (±105 ml)]. Muscle radiodensity was significantly higher following CM administration [Non-contrast: 51.1 HU (±1.9 HU), e.g., Late: 56.6 HU (±2.4 HU), *p* < 0.001]. The human validation analysis showed similar tendencies for the IMAT volume [Non-contrast: 1750ml (±729 ml), Venous: 1,552 ml (±696 ml), *p* = 0.10] and significantly higher muscle radiodensity [Venous: 39.2 HU (±9.1 HU), Non-contrast: 35.6 HU (±7.8), *p* = 0.007].

**Conclusion:**

Myosteatosis surrogates, such as muscle radiodensity or IMAT, are susceptible to interference by CM, while quantification of muscle tissue and extramuscular fat remains robust.

## Introduction

Computed tomography (CT) is a pivotal tool in modern medical care, routinely used for the diagnosis and follow-up of various diseases. CT-based body composition analysis (BCA) has advanced from manual or semi-automatic segmentations to an accurate and fully automated 3D tool, which can be easily integrated into clinical workflows (1, 2). Prior studies, using functional and anthropometric surrogates such as hand grip strength or bioelectrical impedance analysis, have associated skeletal muscle loss and myosteatosis to significantly worse survival outcomes in both chronically ill and healthy elderly patients (3–5). For instance, Chuang et al. demonstrated that healthy participants in the lowest quartile of the skeletal muscle index (skeletal muscle mass divided by height squared) in a national health survey had a significantly higher overall and cardiovascular mortality (5). Over the past years, image-based biomarkers—such as sarcopenia or myosteatosis—extracted by BCA from routine imaging have been thoroughly investigated for risk stratification or opportunistic screening. At the disease baseline or before an intervention, e.g., surgery, those results have been linked to numerous outcomes like disease severity, overall survival, or length of hospital stay (6–11). In patients with metastatic renal cancer, an increased share of subcutaneous adipose tissue (SAT) was associated with a significantly improved progression-free and overall survival (9). Before surgery, lower muscle mass with and without concurrent obesity was significantly associated with a longer hospital stay and an increased complication rate in abdominal and thoracic surgery (7, 12).

Currently, there are only a few studies that evaluate the dynamics of evolving cachexia and sarcopenia. Lee et al. demonstrated that a reduction of SAT within two months after the start of chemotherapy was an independent risk factor for overall survival in patients with metastatic pancreatic cancer (13). Those results highlight the potential of surveilling BCA in chronic diseases, and further longitudinal studies may unveil novel metrics for therapy monitoring or even interventions. Robust results under different scan conditions and seamless integration in care pathways are mandatory parameters for efficient BCA usage.

Recently, Haubold *et al*. pioneered the seamless integration of fully automated BCA in the clinical routine via a DICOM node in their open-source Body and Organ analysis (BOA) (2). Like most BCA networks, tissue classification by the BOA relies on a combination of modern segmentation techniques and radiodensity [Hounsfield Unit (HU)] thresholding for sub-segmentation within determined regions (2, 14, 15). Thus, an alteration in radiodensity following CM administration could be a relevant confounder for longitudinal monitoring.

Some studies have shown that intravenous contrast administration can affect CT-based body composition metrics, particularly adipose-tissue-related measurements (16, 17). For instance, Troschel et al. observed lower SAT, visceral adipose tissue (VAT), and intermuscular adipose tissue areas after CM-injection (16). For the muscle area, only smaller deviations of 2.4% were documented (17). Still, there is a lack of studies on the impact of CM on volumetric BCA-networks. Accordingly, the effect of CM on state-of-the-art volumetric pipelines remains insufficiently characterized. Additionally, CT contrast phases can vary substantially both between and within individuals, with circulation being the primary driver alongside other influences (18). As a result, phase assignment may differ markedly—for example, a scan classified as vascular portal venous in one patient may correspond more closely to a late arterial phase in another—while more subtle distinctions, such as between early and late arterial phases, may also be challenging.

Therefore, we included a controlled large-animal model in our study to enable repeated within-subject acquisitions across multiple contrast phases under highly standardized scan, injection, and physiological conditions with a high time resolution. Thereby, we aimed to minimize variations inherent to human data and to isolate the specific effect of contrast administration on BCA. To translate into clinical imaging this study will combine the large-animal study in laboratory conditions with real-world patient data to evaluate the accuracy of the previously published open-source BOA network across multiple phases following the intravenous injection of CM.

## Materials and methods

### Ethical considerations

The animals were investigated in compliance with the German Animal Welfare legislation (Landesamt für Gesundheit und Soziales, Berlin, Germany). The study was approved by the State Animal Welfare Committee. The human analyses were conducted in concordance with the local Institutional Review Board's guidelines (approval number: 23-11410-BO). Written informed consent was not required in accordance with institutional requirements and national legislation. All data were fully anonymized before inclusion.

### Animals

Göttingen minipigs were chosen as the animal model because of their size and anatomy. The abdominal cross-section of the animals (27 cm) is comparable to abdominal diameters reported in human clinical imaging studies (19). Additionally, Göttingen minipigs are a well-established large animal model for CT and CM research and the multiphasic CT imaging model is a well-established methodology in our lab (20–24). As this study is an additional investigation of parts of a previously conducted study, no animal was investigated exclusively to work on this hypothesis (24, 25). The scans of the twenty Göttingen minipigs (Ellegaard, Dalmose, Denmark) were performed under general anesthesia. After intramuscular injection of azaperone [2 mg/kg (Stresnil; Elanco GmbH, Bad Homburg, Germany)], ketamine [15 ml/kg (Pharmacia, Karlsruhe, Germany)], and intravenous administration of 7 mg/kg propofol (Propofol-Lipuro; B. Braun, Melsungen, Germany), the animals were orally intubated and mechanically ventilated with an air-oxygen mixture. Anesthesia was maintained with an intravenous propofol infusion of 12 mg/kg/h. The vital parameters of the animals were consequently monitored.

### Computed tomography imaging

Porcine CT images were acquired in the prone position under end-expiratory ventilation stop on a 192-slice dual-source CT scanner (Somatom Force, Siemens Healthineers, Erlangen, Germany). Scans were conducted before the injection of contrast medium and during various CM-phases: Early Arterial phase [Early Arterial; 19 s post-injection (p.i.)], Late Arterial phase (Late Arterial; 24 s p.i), Vascular Portal Venous phase (Vascular Portal Venous; 48 s p.i.), Parenchymal Portal Venous phase (Parenchymal Portal Venous; 68 s p.i.), Late phase (180 s p.i.). On all porcine scans, Scan parameters were the following: 0.5 s rotation time, 0.6 pitch, 150 mm scan length (starting at the cranial liver border on the scout view), 90 kV tube voltage, 350 mAs tube current-time product. Reconstruction parameters were the following: 1-mm slice thickness, 300 × 300 mm field of view, Br40 kernel, and SAFIRE 3 iterative reconstruction. A Medrad Centargo CT injection system (Bayer AG, Berlin, Germany) was utilized for the administration of the contrast medium [350 mg l/kg iopromide (Ultravist 300; Bayer Vital GmbH, Leverkusen, Germany)]. Following this, a 20 mL saline bolus was given at the same flow rate.

### Study design

A total of 60 CT examinations of the abdomen were conducted on the twenty Göttingen minipigs. These examinations were spread across three separate sessions, grouped into three blocks (repetitions). Between each block, there an at least an eight-week gap. Due to incomplete scans, one exam was eliminated from the study, resulting in a total of 59 studies.

### Body composition analysis

The previously described network BOA network, employing a multi-resolution variant of the nnU-Net architecture, was utilized to extract body composition features. This network facilitates automated tissue segmentation within designated body regions in CT scans, achieving high accuracy in segmentation (2). Similar to other BCA models, the model used in this study integrates modern segmentation techniques along with HU-thresholding to distinguish specific tissue types (adipose tissue, muscle tissue). In the predefined BOA network, tissue classification is initiated by predicting several body region classes via a deep-learning model. Among those classes are: subcutaneous tissue, muscle and abdominal cavity (2). Sub-segmentation relies on quantification metrics first described in the BCA-network by Koitka et al., with densities between −190 and −30 HU for fat tissue and −29 and 150 HU for muscle tissue (2, 14, 26, 27). For instance, thresholding for fat isodense voxels in the visceral compartment would result in the label VAT, and in the muscular compartment, it would result in IMAT (2, 14).

**Figure 1:**
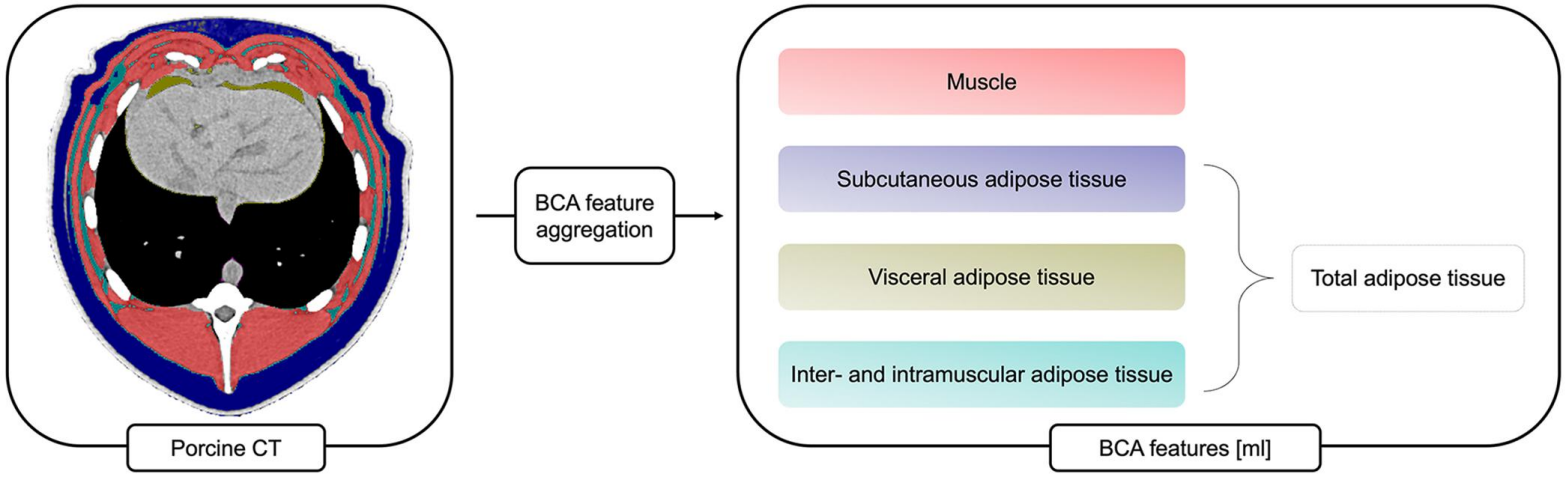
BCA feature extraction. Different BCA features that depend on segmentation algorithms and HU-thresholding are extracted by the network. The tissues are coded as follows: red: Muscle, dark blue: Subcutaneous adipose Tissue, yellow: Visceral adipose tissue, light blue: Inter- and Intramuscular adipose tissue. BCA, body composition analysis.

The following features rely on both mechanisms and were extracted as volumes in milliliters (ml) according to the output convention of the BOA pipeline for volumetric measurements: Muscle, Subcutaneous adipose tissue (SAT), Visceral adipose tissue (VAT), and Intra- and intermuscular adipose tissue (IMAT). Total adipose tissue (TAT) volume represents the sum of the different fat tissue subtypes (SAT, VAT, IMAT). Muscle radiodensity was given as the mean attenuation of all voxels within the muscle label of the BOA network, expressed in HU (Figure 1).

### Control segmentation and validation of the automated BCA

The BOA network was trained and validated on human datasets (2). Two radiology residents supervised by a consultant radiologist with 8 years of experience in abdominal imaging manually annotated every 25th slice (slice thickness and increment of 1 mm) on 30 CT examinations (5× per CM phase) to analyze the reliability in the animal model. The Dice Score assessed the overlap of the fully automated segmentation and the manual control (28):

$$DICE\;(A,\;C)=\frac{2\times |A\cap C|}{|A|+|C|}$$

A and C represent the fully automatically generated (A) and manually segmented (C) sets of images. ∣A∣ & ∣C∣ denote as the respective sample size, while ∣A∩C∣ stands for the intersection between the two groups.

### Human validation cohort

Initially, a Fast Healthcare Interoperability Resource-based query for clinically indicated, tri-phasic CT angiographic scans of the entire Aorta in a single tertiary-care centre resulted in 173 exams from 10/2023 to 10/2024. Examinations with a missing non-contrast scan of the abdomen (*n* = 36), major artifacts (*n* = 11), slice thickness of >1 mm in at least one phase (*n* = 3), and incomplete coverage of the abdominal cavity in at least one phase (*n* = 9) had to be excluded. This resulted in a final cohort of 114 tri-phasic CT scans. Characteristics of the human validation cohort are summarized in Table 1.

**Table 1:** Patient characteristics of the human validation cohort.

Variable	Human Validation Cohort *(n* *=* *114)*
Age, years (mean ± SD)	63 (±13)
Female sex, *n* (%)	40 (35%)
Outpatients, *n* (%)	69 (61%)
Clinical indication categories	
Suspected acute aortic pathology, *n* (%)	55 (48%)
Pre- or post-interventional workup, *n* (%)	48 (42%)
Aneurysm surveillance, *n* (%)	3 (3%)
Other, *n* (%)	8 (7%)

The scan protocol involved an Arterial (triggered with bolus tracking at 120 HU in the ascending Aorta) and a Venous phase (50 s after the Arterial phase) after an initial Non-contrast scan. All scans were performed on a 192-slice dual-source CT scanner (Somatom Force, Siemens Healthineers, Erlangen, Germany). To optimize the comparability to the porcine scans, the BOA analysis of the human validation was limited to the abdominal cavity with the exclusion of the extremities. For details on the scan parameters, see Supplementary Material S1.

### Statistical analysis

A D'Agostino-Pearson test confirmed normal distribution of the data. Repeated measures one-way ANOVA with Tukey's multiple comparison tests determined statistical significance. For the intra-individual analysis, the respective CM-phases value [Early Arterial, Late Arterial, Vascular Portal Venous, Parenchymal Portal Venous, Late [animal model]; Arterial, Venous [human validation]] was referred to the Non-contrast value and normalized for percent: $Diff(\text{\%})=\frac{CM\;(ml)-NC\;(ml)}{NC\;(ml)}\;\times \;100\text{\%}$. Normally distributed data is given as mean with standard deviation (±SD). The significance level was set at *p* = 0.05, and all analyses were performed and displayed with GraphPad Prism (10.2.3) for MacOS. L.S. serves as the statistical guarantor of the study.

## Results

### BCA evaluation in the animal model

**Figure 2:**
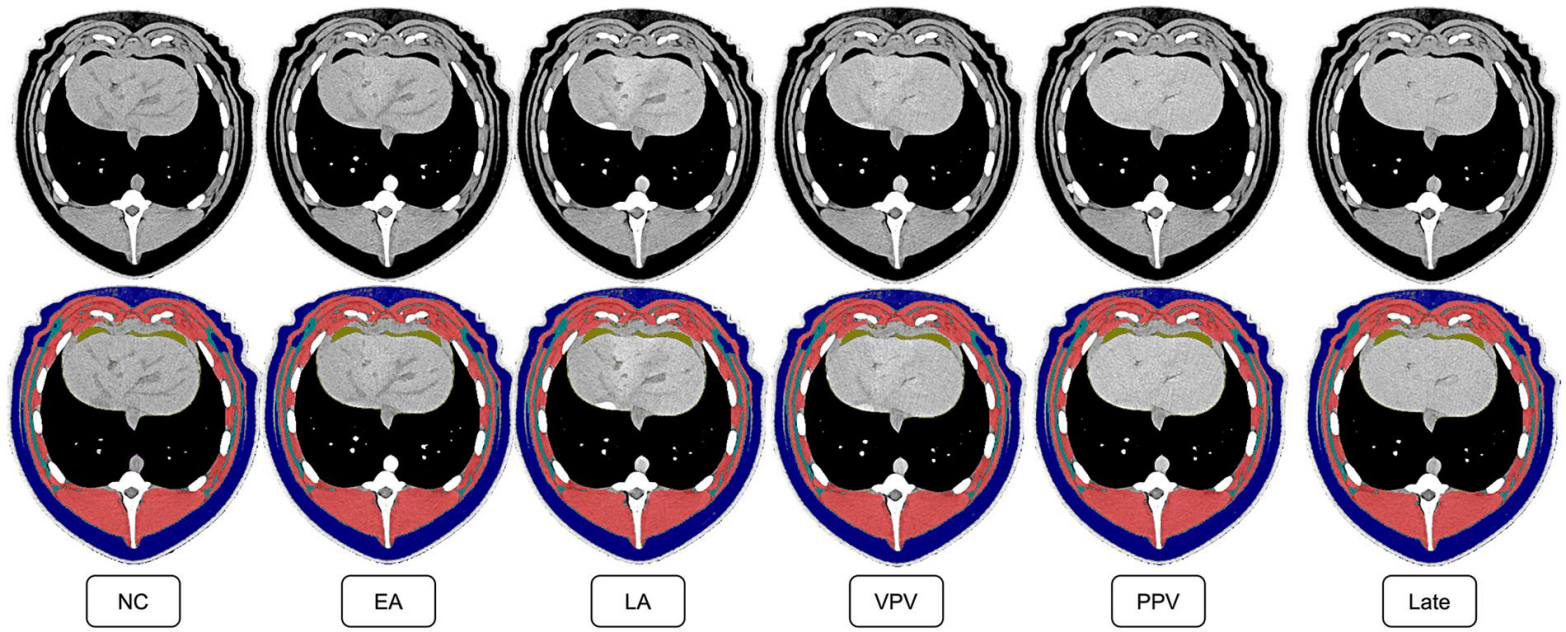
CT slices on the same level in the different CM phases, both without and with annotation of the BCA features. Axial CT slices on the thoracoabdominal junction with and without the segmentation of the BCA features in the different CM phases. The tissues were coded in color as follows; red: Muscle, dark blue: Subcutaneous adipose tissue, yellow: Visceral adipose tissue, light blue: Inter- and intramuscular adipose tissue. NC, non-contrast; EA, early arterial; LA, late arterial; VPV, vascular portal venous; PPV, parenchymal portal venous.

To ensure the porcine applicability of a primarily human-trained network, spatial overlap analysis was performed. The overall Dice score for the overlap of fully automated segmentation to manual controls was 0.883 (Dice_ovr_). In the analysis of the different CM phases, no relevant distortion regarding the respective Dice Score was observed compared to the entire cohort (Dice_NC_ = 0.876, Dice_EA_ = 0.893, Dice_LA_ = 0.886, Dice_VPV_ = 0.868, Dice_PPV_ = 0.889, Dice_Late_ = 0.887) (Figure 2).

### Quantitative BCA results of the animal model

Muscle volumes remained highly consistent across all scans. Absolute measurements were nearly identical between the non-contrast phase [1,376 ml (±149 ml)] and contrast-enhanced phases (ranging from 1,373 ml to 1,390 ml). The minimal relative deviations [e.g., Early Arterial at −0.25% (±0.46%)] and Late at 1.03% (±0.73%) support that consistency. IMAT volumes exhibited no significant differences either. Yet, the contrast-enhanced phases tended to show slightly lower absolute IMAT values compared to the non-contrast phase [e.g., Late Arterial 239 ml (±103 ml) vs. Non-contrast 245 ml (±105 ml)], with minor corresponding relative deviations [e.g., Early Arterial = −2.30% (±1.26%)].

**Figure 3:**
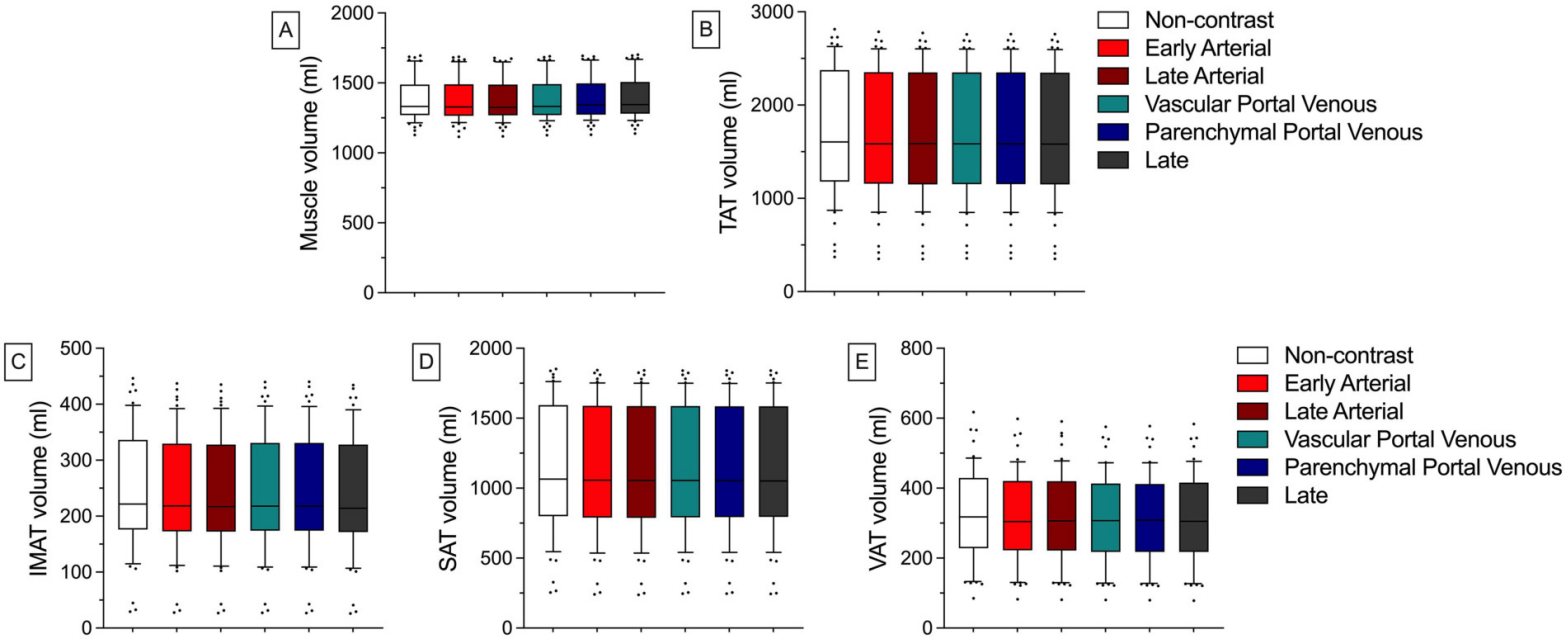
Absolute quantitative BCA results of the animal model in different CM phases. There are no significant differences between the different CM phases for any BCA feature **(A-E)**. Whiskers represent the 10th and 90th percentiles. Dots represent individual values. IMAT, inter- and intramuscular adipose tissue; SAT, subcutaneous adipose tissue; TAT, total adipose tissue; VAT, visceral adipose tissue.

Likewise, no significant differences in the measured volumes were observed across all remaining BCA features. For all absolute volumes and relative deviations, refer to Supplementary Material S2, S3 (Figure 3).

### Human validation cohort characteristics

The human validation cohort comprised 114 patients with a mean age of 63 (±13) years, of whom 40 (35%) were female. Most examinations were performed in the outpatient setting (69/114, 61%). The main clinical indication categories were suspected acute aortic pathology (55/114, 48%) and pre- or postinterventional workup (48/114, 42%). Aneurysm surveillance (3/114, 3%) and other indications (8/114, 7%) were less frequent (Table 1).

### Quantitative BCA of the human validation cohort

**Figure 4:**
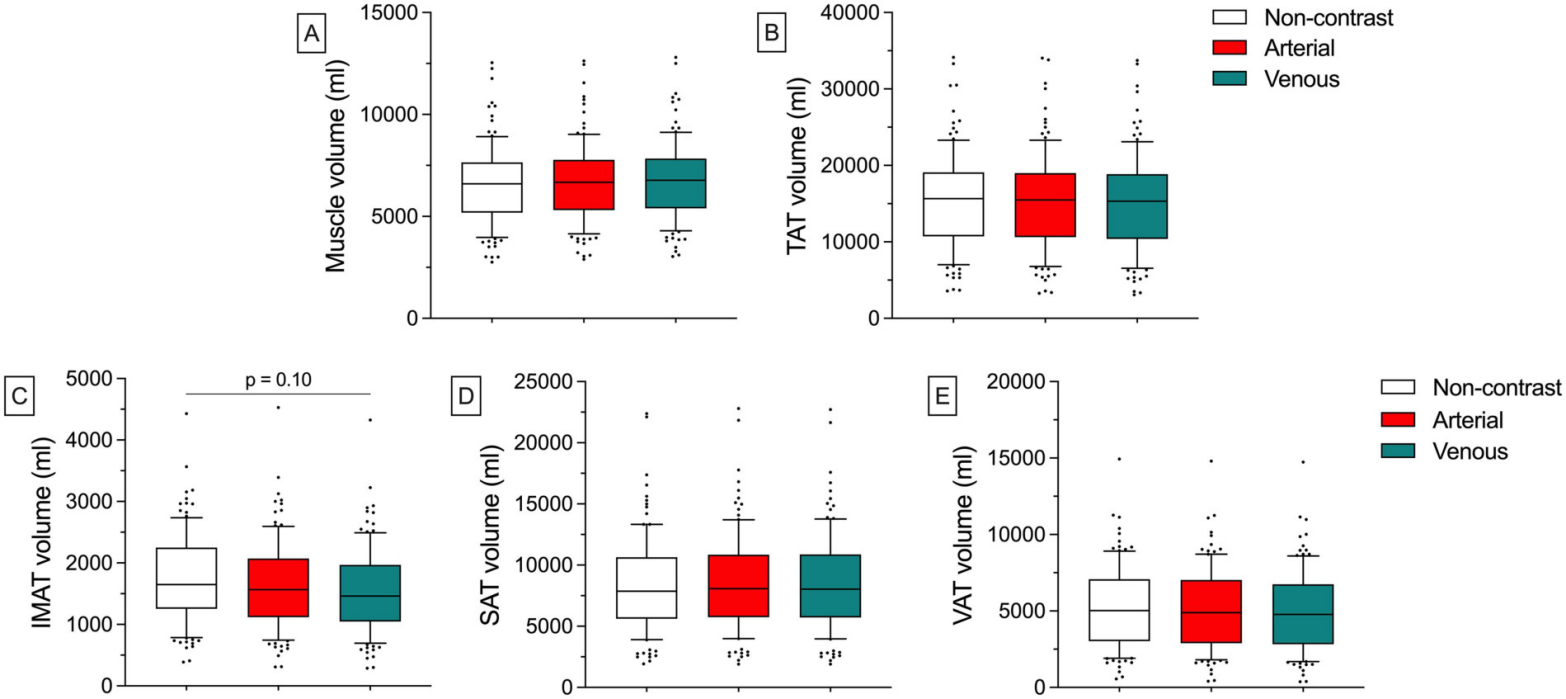
Absolute quantitative BCA results of the human validation cohort in different CM phases. After the injection of CM, IMAT volumetry was lower than in the Non-contrast scan [e.g., Venous phase: 1,552 ml (±696 ml) vs. Non-contrast: 1750 ml (±729 ml), *p* = 0.10; (C)]. There are no significant differences for the other features **(A, B. D. E)**. Whiskers represent the 10th and 90th percentiles. Dots represent individual values. IMAT, inter- and intramuscular adipose tissue; SAT, subcutaneous adipose tissue; TAT, total adipose tissue; VAT, visceral adipose tissue.

In the tri-phasic CT scan of the human validation cohort, IMAT decreases from the Non-contrast [1750ml (±729 ml)] to the Arterial [1,643 ml (±723 ml)] and Venous phase [1,552 ml (±696 ml), *p* = 0.10] with mean intraindividual deviations of −6.93% (±6.61%) in the Arterial and −12.4% (±6.43%) in the Venous phase. In comparison to that, the remaining BCA features remained stable after the injection of CM. The Muscle volume increased from 6,571 ml (±1951ml) to 6,685 ml (±1,950 ml) in the Arterial phase and 6,765 ml (±1925 ml) in the Venous phase. For all absolute volumes and relative deviations of the BCA features, refer to Supplementary Material S4, S5 (Figure 4).

### Muscle radiodensity analysis

**Figure 5:**
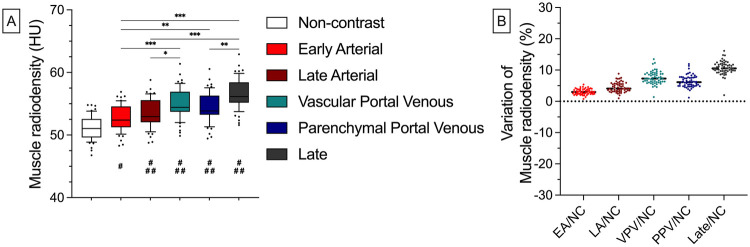
Absolute results and relative deviations of porcine muscle radiodensity. Muscle radiodensity significantly increased from 51.1 HU (±1.9 HU, NC) to 52.7 HU (±2.0 HU, *p* = 0.04; EA), 53.4 HU (±2.2 HU, *p* < 0.001; LA), 55.0 HU (±2.4 HU, *p* < 0.001; VPV), 54.4 HU (±2.4 HU, *p* < 0.001; PPV) and 56.6 [±2.4, *p* < 0.001; Late; **(A)**]. Relative deviations reached from 3.1% (±0.9%) in the Early Arterial to 10.7% (±2.1%) in the Late phase **(B)** Whiskers represent the 10th and 90th percentiles. Dots represent individual values. Horizontal, black, and dashed lines stand for the median. * = *p* < 0.05, ** = *p* < 0.01, *** = *p* < 0.001; ## = *p* < 0.01 compared to NC, ### = *p* < 0.001 compared to NC. NC, non-contrast; EA, early arterial; LA, late arterial; VPV, vascular portal venous; PPV, parenchymal portal venous; IMAT, inter- and intramuscular adipose tissue; SAT, subcutaneous adipose tissue; TAT, total adipose tissue; VAT, visceral adipose tissue.

In the porcine animal model, Muscle radiodensity was significantly higher in the contrast-enhanced scans [e.g., Parenchymal Portal Venous phase: 54.4 HU (±2.4 HU) vs. Non-contrast: 51.1 HU (±1.9 HU); *p* < 0.001]. Similarly, mean deviations compared to the Non-contrast scan increased after CM administration [e.g., Vascular Portal Venous: 7.6% (±2.1%)]. Additionally, there were significant differences between the different contrast-enhanced scans. For instance, Muscle radiodensity was significantly higher in the Vascular Portal Venous phase [55.0 HU (±2.4 HU)] compared to the Early Arterial phase 52.7 HU (±2.0 HU, *p* < 0.001). For all density values and relative deviations, refer to Supplementary Material S6 (Figure 5).

**Figure 6:**
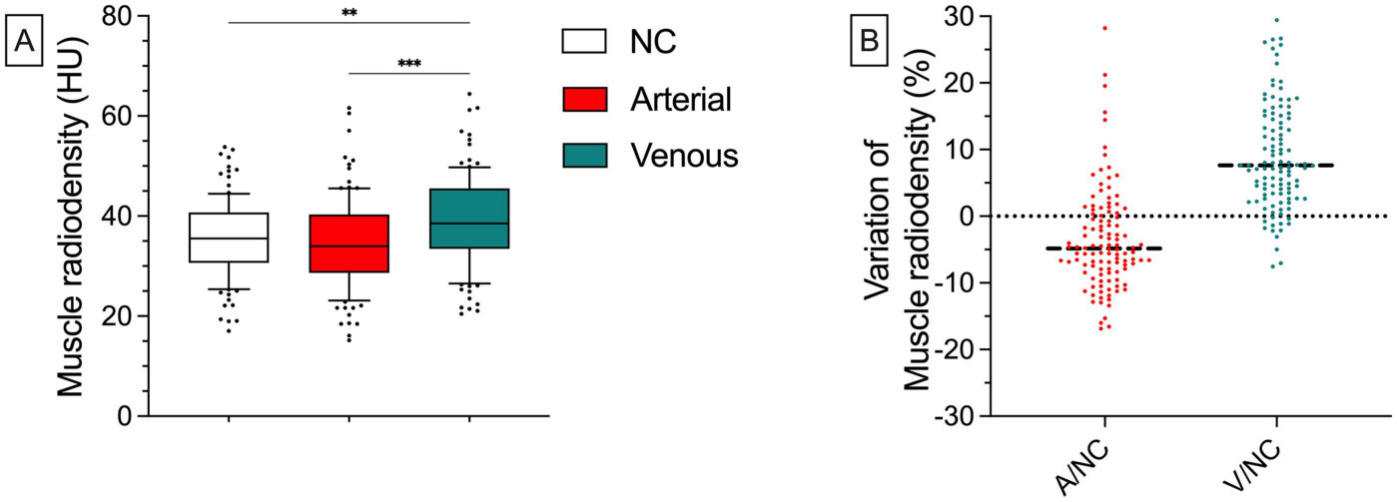
Absolute results and relative deviations of human muscle radiodensity in different CM phases. After the administration of CM, there is a tendency to lower Muscle radiodensity in the Arterial phase [34.6 HU (±9.0 HU)] and significantly higher values in the Venous phase [39.2 HU (±9.1 HU), *p* = 0.007] compared to the Non-contrast phase (35.6 HU [±7.8 HU; **(A)**]. The mean intraindividual deviation from the Non-contrast scan was −3.5% (±7.6%) in the Arterial phase and 10.0% (±9.7%) in the Venous phase **(B)** Whiskers represent the 10th and 90th percentiles. Dots represent individual values. Horizontal, black, and dashed lines stand for the median. A, arterial; NC, non-contrast; V, venous.

In the human validation cohort, muscle radiodensity was significantly higher in the Venous phase [39.2 HU (±9.1 HU)] compared to both, the Non-contrast scan [35.6 HU (±7.8 HU), *p* = 0.007] and the Arterial phase [34.6 HU (±9.0 HU), *p* < 0.001]). No significant differences were observed between the Non-contrast scan and the Arterial phase. The mean intraindividual deviation from the non-contrast scan was −3.52% (±7.61%) in the Arterial phase and 10.0% (±9.7%) in the Venous phase. For all density values and relative deviations, refer to Supplementary Material S7 (Figure 6).

## Discussion

In this translational study, intravenous CM administration had minimal impact on volumetric muscle and extramuscular adipose tissue quantification but affected myosteatosis-related parameters. While Muscle, SAT, VAT, and TAT remained largely stable across all CM-phases, IMAT tended to decrease after contrast injection, and muscle radiodensity increased significantly, particularly in later phases. The human validation cohort confirmed this overall pattern, with significantly higher muscle radiodensity and a similar tendency toward lower IMAT values in the venous phase. These findings suggest that BOA-derived markers of muscle quantity are comparatively robust across contrast phases, whereas muscle quality surrogates should not be compared interchangeably between non-contrast and contrast-enhanced CT examinations.

CT-based BCA is in the spotlight of current radiological research as it has proven to deliver biomarkers for the stratification of survival probability and several adverse events (7–11). Besides muscle volume, pathological fat infiltration in muscles and accumulation around them—myosteatosis—is associated with decreased muscle quality and function and has been proven to be a risk factor in diseases and among healthy elderly individuals (29–31). In CT, myosteatosis is most frequently measured by a reduction of muscle radiodensity, which itself is known to be influenced by CM (32, 33). In previous studies in certain regions of interest and on full CT-slices, muscle density increased up to 57% in a venous and 43% in a delayed contrast phase (34, 35). Our study underscores this inconsistency at a lower scale, with an increase of 6.4% in the Parenchymal Portal Venous and 10.7% in the Late phase compared to the non-contrast radiodensity of porcine muscle tissue. In contrast, IMAT volume showed only a minor reduction of up to −3.43% (±1.98%) in the animal model. But differences of −12.4% (±6.43%) were observed in the venous scan of the human validation cohort, highlighting the instability of IMAT volumetry after CM injection. Particularly, the results of the human validation cohort align with the study by Troschel et al., who observed a reduction of −9.3% in IMAT using semi-automatic single-slice segmentation at the level of L3 during the portal venous phase (16). With mean deviations of less than 1.03% in the animal model and 3.43% in the human validation, muscle quantification was largely unaffected by CM and appears comparatively robust between different contrast phases. This enables a broader implementation in imaging datasets with mixed contrast phases and longitudinal comparison of the same patients.

Adipose tissue, long considered merely an energy storage depot, is now recognized as a dynamic endocrine organ with far-reaching effects on human health (36, 37). CT-based BCA, is becoming the reference standard for precise body fat distribution analysis, as other measurements like body mass index or even dual x-ray absorptiometry have significant drawbacks (38). In the clinical context, potential effects of CM have limited the routine implementation and longitudinal comparison of those features as Perez et al. raised concerns about the stability of those features. They reported a mean reduction of 25.4% in VAT area and 9.4% in SAT area on contrast-enhanced scans (17). In our porcine animal model, SAT shows remarkable consistency with deviations of less than 1.1% across all contrast phases. Even in the human validation, deviations of less than 2% underscore this consistency. In contrast, VAT exhibits deviations of up to −3.60% in the late phase of the animal model and of up to −4.73% in the venous phase of the human validation. Nevertheless, those deviations are not statistically significant, and the volumetric results are notably more consistent compared to those in the planimetric study by Perez et al. (17). They do align more closely with findings of Troschel et al., who observed stable SAT measurements and slightly higher deviations in VAT, with a mean change of −2.0% (16). The more pronounced changes in the visceral, compared to the subcutaneous compartment, are likely due to increased radiodensity at the margins of contrast-enhanced vessels, causing voxels to fall outside the fat tissue HU threshold (2).

To tackle those challenges of—major or minor—inconsistencies in BCA, several studies postulate a correction factor for the comparison of Non-contrast and contrast-enhanced scans (16, 17). However, the example of myosteatosis shows that due to the widespread deviations, both in the measurement of Hounsfield units (venous range: −7.5–50.2%) and in the assessment of IMAT (venous range: −31.5–11.6%) in the human validation, the applicability of a single corrective factor for all patients seems impractical. Still, several strategies may help overcome contrast-related limitations of BOA-derived imaging markers. First, longitudinal studies should use a uniform acquisition phase across all examinations. Second, phase-specific reference values or cutoffs, especially for muscle radiodensity, may enable meaningful interpretation of contrast-enhanced scans. Third, phase-aware calibration approaches could improve the robustness of highly HU-dependent biomarkers. Until such methods are validated, myosteatosis metrics should not be compared across mixed contrast phases.

Although we can present interesting results, a few limitations must be discussed thoroughly. The scans of the animal experiment were performed under optimal conditions in exact accordance with all scan variables. To analyze transferability into clinical reality, a human validation cohort of real-world clinical scans was added to account for variations in patients’ anatomy and physiology, CM volume, and dose parameters. Additionally, we investigated a volumetric BCA network incorporating whole scan volumes and not single slices, which might limit the comparability to studies focusing on planimetric and region-of-interest-based measurements. HU thresholds can differ across BCA networks. Within the BOA workflow used in this study, VAT was quantified using a predefined HU threshold range of −190 to −30 HU. However, as VAT HU threshold conventions differ across body composition analysis frameworks, direct translation or comparison of the present findings beyond the implemented BOA pipeline should be made with caution.

Finally, our large-animal study with a human validation cohort demonstrates that the investigated BOA-network for BCA consistently delivers accurate results for muscle quantity and the volumes of visceral and subcutaneous fat. However, myosteatosis, as a surrogate marker for muscle quality (whether measured by radiodensity or IMAT), is influenced by CM administration, making its comparison between different CM phases obsolete.

## Data Availability

The raw data will be made available upon reasonable request to the corresponding author.
