# Supplementary material for: Intravenous contrast medium impairs CT-based muscle quality but not quantity assessment: a translational study
Source: Front Radiol. 2026 May 8;6:1746296. doi: 10.3389/fradi.2026.1746296 (PMC13195011; doi:10.3389/fradi.2026.1746296)
Supplement: Supplementary file 1 [file Datasheet1.docx]

Intravenous contrast medium impairs CT-based muscle quality but not quantity assessment: a large-animal study with human validation

### Supplementary Material 1: Human validation CT acquisition parameters.

|  | **Non-contrast** | | **Arterial** | | **Venous** | |  |  |  |
| --- | --- | --- | --- | --- | --- | --- | --- | --- | --- |
|  |  |  |  | |  | |  | |  |
| Exposure time (s) | 252 (±22) | | 255 (±32) | | 255 (±32) | |  |  |  |
| Pitch factor | 0,6 (±0,2) | | 1,6 (±0,3) | | 1,6 (±0,3) | |  |  |  |
| Scan lengths (cm) | 68 (±5,8) | | 69 (±7,0) | | 69 (±7,0) | |  |  |  |
| Tube potential (kV) | 97,5 (6,9) | | 100 (4,2) | | 100 (±4,2) | |  |  |  |
| CTDIvol (mGy) | 3,9 (±2,2) | | 5,2 (±2,6) | | 5,0 (±2,2) | |  |  |  |
|  |  |  |  |  | |  | |  | |

s = second, mm = millimetre, kV = kilovolt, CTDIvol = volumetric CT dose index, mGy = milligray,.

### Supplementary Material 2: Volumetric results of all investigated BCA features in different CM-phases of the animal model and relative deviations compared to Non-contrast scans.

| Feature feature | **NC** | | **EA** | | **LA**  **VPV** | |
| --- | --- | --- | --- | --- | --- | --- |
|  | Absolute (ml) | Rel./NC (%) | Absolute (ml) | Rel./NC (%) | Absolute (ml) | Rel./NC (%) |
|  |  |  |  | |  |  |
| Muscle (ml) | 1376 (±149) | / | 1373 (±149) | -0.25 (±0.46) | 1373 (±147) | -0.20 (±0.52) |
| TAT (ml) | 1720(±671) | / | 1697 (±667)) | -1.50 (±0.83) | 1695 (±666) | -1.67 (±0.94) |
| IMAT (ml) | 245 (±105) | / | 240 (±103) | -2.30 (±1.26) | 239 (±103) | -2.57 (±1.36) |
| SAT (ml) | 1141 (±451) | / | 1134 (±451) | -0.87 (±0.95) | 1132 (±451) | -1.10 (±1.11) |
| VAT (ml) | 323 (±130) | / | 315 (±128) | -2.58 (±1.37) | 316 (±128) | -2.53 (±1.52) |
|  |  |  |  |  |  |  |
| Feature | **VPV** | | **PPV** | | **Late** | |
|  | Absolute (ml) | Rel./NC (%) | Absolute (ml) | Rel./NC (%) | Absolute (ml) | Rel./NC (%) |
|  |  |  |  |  |  |  |
| Muscle (ml) | 1379 (±147) | 0.23 (±0.50) | 1385 (±147) | 0.66 (±0.78) | 1390 (±148) | 1.03 (±0.73) |
| TAT (ml) | 1694 (±665) | -1.66 (±0.70) | 1694 (±665) | -1.64 (±0.73) | 1692 (±666) | -1.84 (±0.97) |
| IMAT (ml) | 241 (±104) | -2.01 (1.32) | 241 (±105) | -1.94 (1.49) | 238 (±104) | -3.43 (±1.98) |
| SAT (ml) | 1133 (±450) | -0.91 (±0.68) | 1133 (±450) | -0.90 (±0.67) | 1132 (±450) | -0.97 (±0.91) |
| VAT (ml) | 312 (±126) | -3.74 (±1.68) | 312 (±126) | -3.81 (±1.71) | 312 (±127) | -3.60 (±1.96) |

#### NC = Non-contrast, EA = Early Arterial, LA = Late Arterial, VPV = Vascular Portal Venous, PPV = Parenchymal Portal Venous, TAT = total adipose tissue, IMAT = inter- and intramuscular adipose tissue, SAT = subcutaneous adipose tissue, VAT = visceral adipose tissue, ml = milliliter

### Supplementary Material 3: Relative intra-individual variations of the BCA features volumetry in different CM-phases compared to the Non-contrast scan.


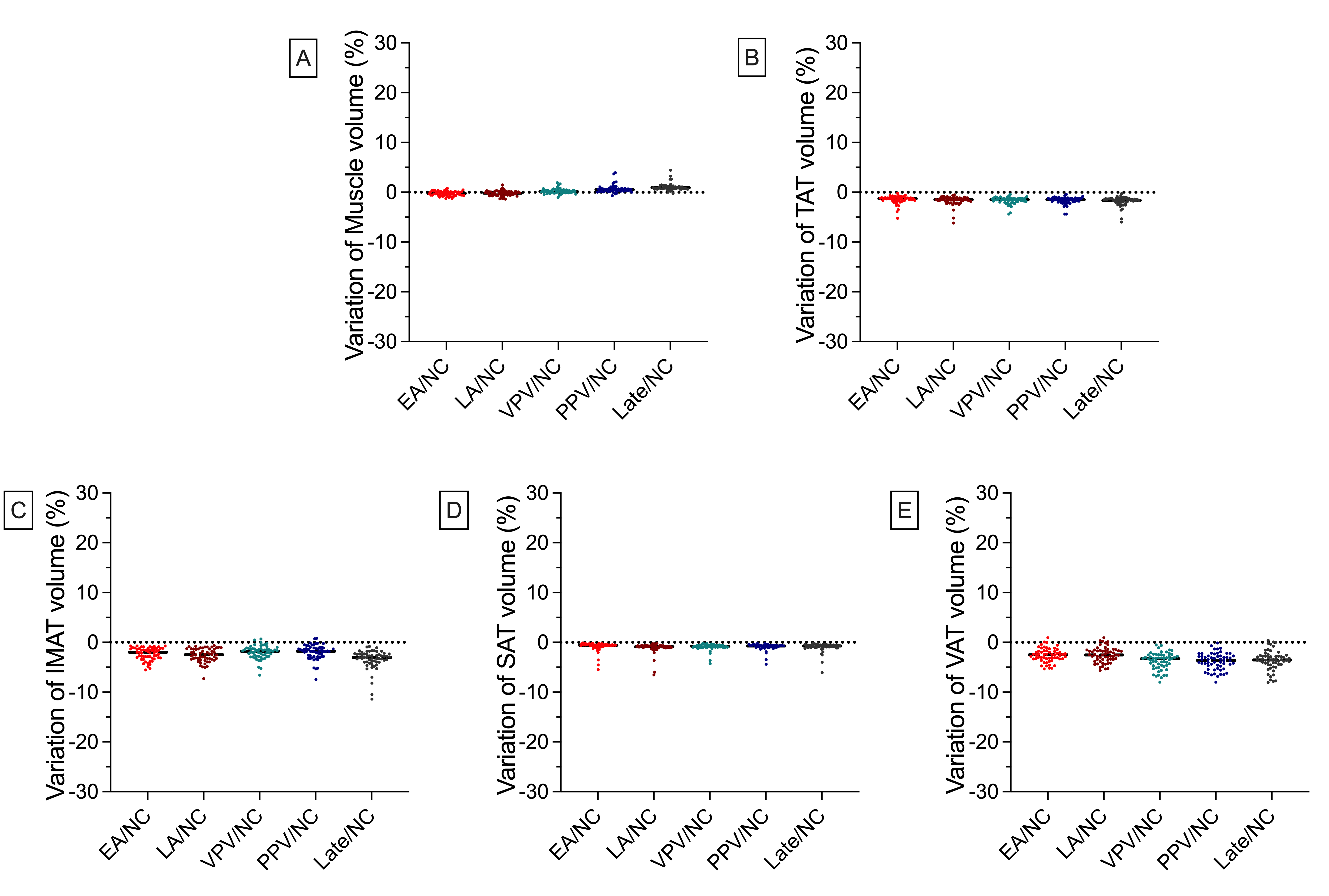


#### Relative deviations of -0.25% (EA), -0.20% (LA), 0.18% (VPV) 0.53% (PPV), and 0.91% (Late) for the Muscle volumetry compared to the Non-contrast scan were observed. Regarding the adipose tissue volumetry, negative deviations ranged from -1.76% (PPV) to -3.05% (Late) for the IMAT and -2.53 % (LA) to -3.60 % (Late) for the VAT volumetry.

#### Dots represent individual values. Horizontal, black, and dashed lines stand for the median.

#### NC = Non-contrast, EA = Early Arterial, LA = Late Arterial, VPV = Vascular Portal Venous, PPV = Parenchymal Portal Venous, IMAT = Inter- and intramuscular adipose tissue, SAT = Subcutaneous adipose tissue, TAT = Total adipose tissue, VAT = Visceral adipose tissue

### Supplementary Material 4: Volumetric results of all investigated BCA features in different CM-phases of the human validation and relative deviations compared to Non-contrast scans.

| Feature feature | **NC** | | **Arterial** | | **Venous**  **VPV** | |
| --- | --- | --- | --- | --- | --- | --- |
|  | Absolute (ml) | Rel./NC (%) | Absolute (ml) | Rel./NC (%) | Absolute (ml) | Rel./NC (%) |
|  |  |  |  | |  |  |
| Muscle (ml) | 6571 (±1951) | / | 6685 (±1950 | 1.95 (±2.31) | 6765 (±1925) | 3.43 (±2.87) |
| TAT (ml) | 15512 (±6422) | / | 15423 (±6479) | -0.94 (±2.11) | 15194 (6470) | -2.75 (±2.66) |
| IMAT (ml) | 1750 (±729) | / | 1643 (±723) | -6.93 (±6.61) | 1552 (±696) | -12.4 (±6.43) |
| SAT (ml) | 8391 (±3909) | / | 8550 (±3979) | 1.87 (±2.35) | 8526 (±3969) | 1.48 (±2.34) |
| VAT (ml) | 5198 (±2696) | / | 5071 (±2698) | -3.65 (±4.53) | 4957 (±2700) | -6.70 (±6.01) |
|  |  |  |  |  |  |  |

#### NC = Non-contrast, A = Arterial, V = Venous, ml = milliliter

### Supplementary Material 5: Relative intra-individual deviations of the BCA metrics in the human validation cohort after the injection of CM


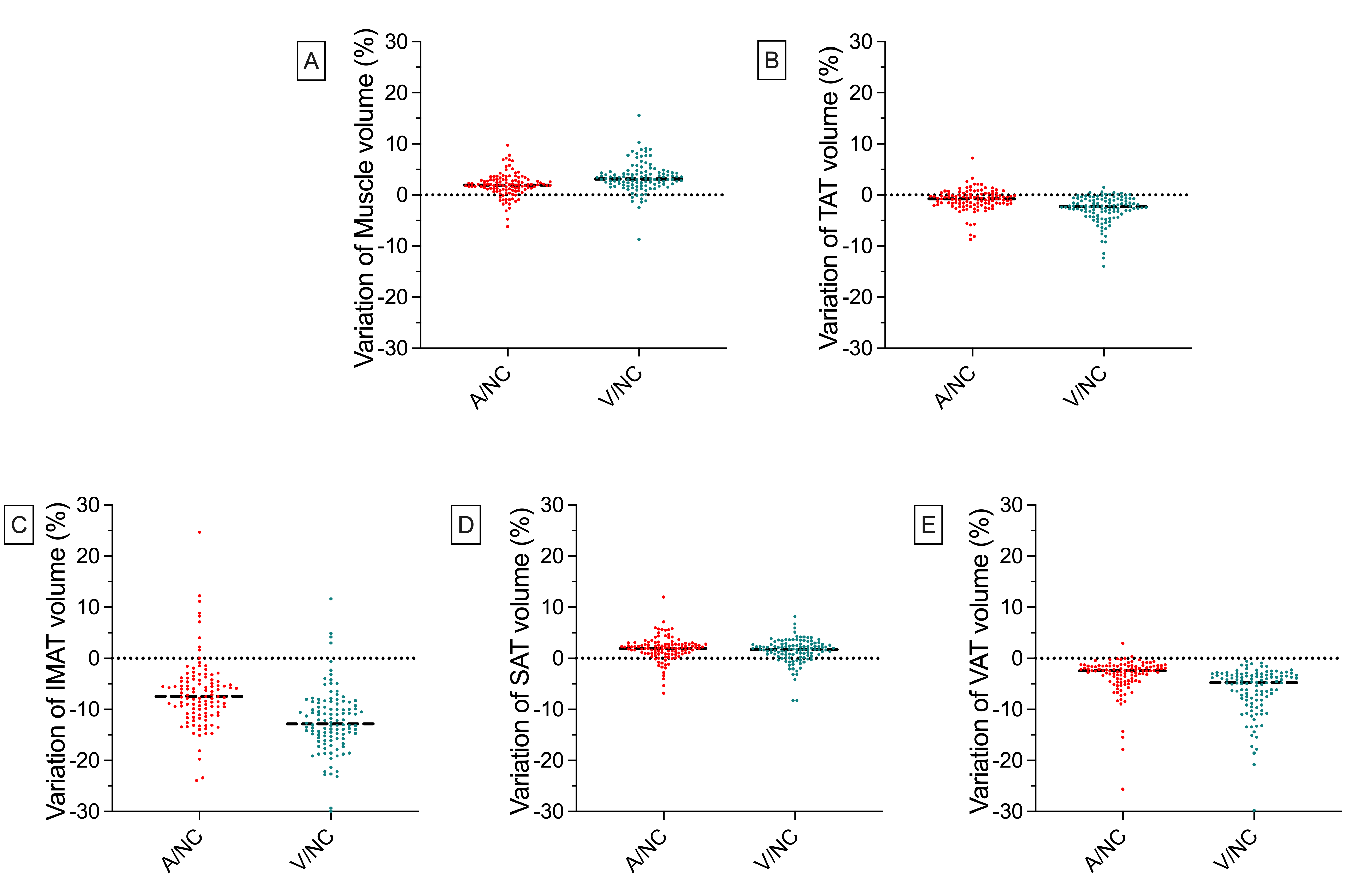


#### In the median, the IMAT volume was -12.8% (-15.7 - -8.87%) smaller in the Arterial phase and -7.46% (-10.8% - -4.29%) smaller in the Venous phase compared to the Non-contrast scan (C). The VAT volume changed by -2.44% (-4.43 - -1.52%) and -4.73% (-8.18% - 3.29%) while the Muscle volume increased by 1.95% (0.98 - 2.96%) and 3.14% (2.01 - 4.39%) in the Arterial and Venous phase, respectively. Dots represent individual values. Horizontal, black, and dashed lines stand for the median. A = Arterial, NC = Non-contrast, V = Venous, IMAT = Inter- and intramuscular adipose tissue, SAT = Subcutaneous adipose tissue, TAT = Total adipose tissue, VAT = Visceral adipose tissue

### Supplementary Material 6: Porcine muscle radiodensity different CM-phases relative deviations compared to Non-contrast scans.

| Feature feature | **NC** | | **EA** | | **LA**  **VPV** | |
| --- | --- | --- | --- | --- | --- | --- |
|  | Absolute (HU) | Rel./NC (%) | Absolute (HU) | Rel./NC (%) | Absolute (HU) | Rel./NC (%) |
|  |  |  |  | |  |  |
| Muscle (ml) | 51.1 (±1.9) | / | 52.7 (±2.0) | 3.1 (±0.91) | 53.4 (±2.2) | 4.5 (±1.6) |
|  |  |  |  |  |  |  |
| Feature | **VPV** | | **PPV** | | **Late** | |
|  | Absolute (ml) | Rel./NC (%) | Absolute (ml) | Rel./NC (%) | Absolute (ml) | Rel./NC (%) |
|  |  |  |  |  |  |  |
| Muscle (ml) | 55.0 (±2.4) | 7.6 (±2.0) | 54.4 (±2.4) | 6.4 (±1.9) | 56.6 (±2.4) | 10.7 (±2.1) |

#### NC = Non-contrast, EA = Early Arterial, LA = Late Arterial, VPV = Vascular Portal Venous, PPV = Parenchymal Portal Venous, HU = Hounsfield Units

### Supplementary Material 7: Human muscle radiodensity in different CM-phases and relative deviations compared to Non-contrast scans.

| Feature feature | **NC** | | **Arterial** | | **Venous**  **VPV** | |
| --- | --- | --- | --- | --- | --- | --- |
|  | Absolute (HU) | Rel./NC (%) | Absolute (HU) | Rel./NC (%) | Absolute (HU) | Rel./NC (%) |
|  |  |  |  | |  |  |
| Muscle (ml) | 35.6 (±7.8) | / | 34.6 (±9.0) | -3.5 (±7.6) | 39.2 (±9.1) | 10.0 (±9.7) |

#### NC = Non-contrast, A = Arterial, V = Venous, HU = Hounsfield Units
